# Primary Extrapulmonary Rifampicin Mono-Resistant Tuberculosis of the Parotid Gland in an Indian Female: The World’s First Case

**DOI:** 10.7759/cureus.33114

**Published:** 2022-12-29

**Authors:** Sankalp Yadav

**Affiliations:** 1 Medicine, Shri Madan Lal Khurana Chest Clinic, Moti Nagar, New Delhi, IND

**Keywords:** drug-resistant tuberculosis, parotidectomy, rifampicin mono-resistance tuberculosis, parotid glands, tuberculosis

## Abstract

Tuberculosis at extrapulmonary sites is relatively uncommon. A case of isolated involvement of the salivary gland is even rare and primary rifampicin mono-resistant tuberculosis of the parotid gland is rarest of rare with no such report ever documented in the medical literature. In this case report, the author presents a case of primary extrapulmonary rifampicin mono-resistant tuberculosis of the parotid gland in a 44-year-old Indian female. She presented with complaints of a painless swelling in the right parotid region and underwent a detailed diagnostic work-up including fine needle aspiration cytology, cartridge-based nucleic acid amplification test, computed tomography scan of head and neck, and culture, and was diagnosed with primary extrapulmonary rifampicin mono-resistant tuberculosis of the parotid gland. The patient was initiated on a WHO-recommended regimen per the national guidelines. After nine months of treatment, she had no symptoms and was declared as treatment completed. With no such case ever reported, this case emphasizes the importance of a high degree of suspicion for rare presentations of common diseases like tuberculosis even in the absence of a history, contacts, or other constitutional symptoms.

## Introduction

Tuberculosis is a bacterial infection caused by* Mycobacterium tuberculosis *and is rampant in countries of Asia, Africa, and Europe [1]. It is a public health issue and the data from the recent Global TB Report of the WHO indicates that for every 0.1 million population, there were 134 cases with a total of 10.6 million people infected in the year 2021 [2]. The data is alarming especially with an increase in the total number of cases reported compared to the year 2020 [2].

Further, the data of drug-resistant tuberculosis i.e., multidrug-resistant/rifampicin-resistant tuberculosis for the year 2021 showed a peak of 3.1% cases compared to the year 2020 [1]. This increase in the number of cases was ultimately associated with substantial mortality (119,000-264,000) for the year 2021 [2]. Rifampin-resistant tuberculosis affected 465,000 individuals worldwide in the year 2019 with 78% multidrug-resistant tuberculosis and 22% rifampicin mono-resistant tuberculosis, respectively [3].

Extrapulmonary tuberculosis is comparatively rare and the diagnosis, particularly in the absence of any pulmonary involvement, medical history, contact with a tuberculosis case, or at relatively rare sites like salivary glands, is a formidable task [4]. It requires a very high index of suspicion backed with investigations like fine needle aspiration cytology (FNAC), cartridge-based nucleic acid amplification test (CBNAAT), computed tomography (CT), and culture to diagnose tuberculosis in paucibacillary cases of parotid swelling. A case of primary extrapulmonary rifampicin mono-resistant tuberculosis of the parotid gland is presented here with no history of tuberculosis or any contact. A detailed literature search revealed that there is a paucity of literature related to rifampicin mono-resistant tuberculosis in the salivary glands like the parotid gland which makes the present case unique as for the first time resistance to rifampicin is identified from the sample from a swollen right parotid gland.

## Case presentation

In February 2021, a 44-year-old Indian female belonging to a middle-income family came to the outpatient department with complaints of painless swelling on the right side of her face. The swelling was initially very small but evolved to around 5 cm in size during the last three months. There were no complaints of fever, cough with or without expectoration, night sweats, or loss of appetite/weight. There was no history of tuberculosis in her or any of her contacts. Further, there was no history of substance abuse or imprisonment.

General examination revealed an afebrile patient, with a pulse of 88 per minute, blood pressure of 125/82 mm of Hg, respiratory rate of 19 per minute, oxygen saturation (SpO_2_) of 94% on room air, and weight of 75 kg with a body mass index (BMI) of 27.55 kg/m^2^. There was no pallor, cyanosis, edema, lymphadenopathy, or icterus. Local examination revealed a single, nearly 5 cm x 3 cm irregular swelling on the right side of the face i.e., at the right parotid region in front of the right ear. The surface of the swelling was nearly smooth with no abnormalities on the overlying surface of the skin. There was no erythema or raised temperature over the swelling. The swelling was firm, non-tender, and mobile. There was no similar swelling on the opposite side of the face. The facial nerve function was intact. Her systemic examination was unremarkable.

Based on the clinical picture, a primary diagnosis of parotitis under investigation was made with differentials such as parotid gland stones, parotid cysts, collagen vascular disorders like sarcoidosis, dental problems, and viral and bacterial infections like tuberculosis, and malignancy.

**Figure 1:**
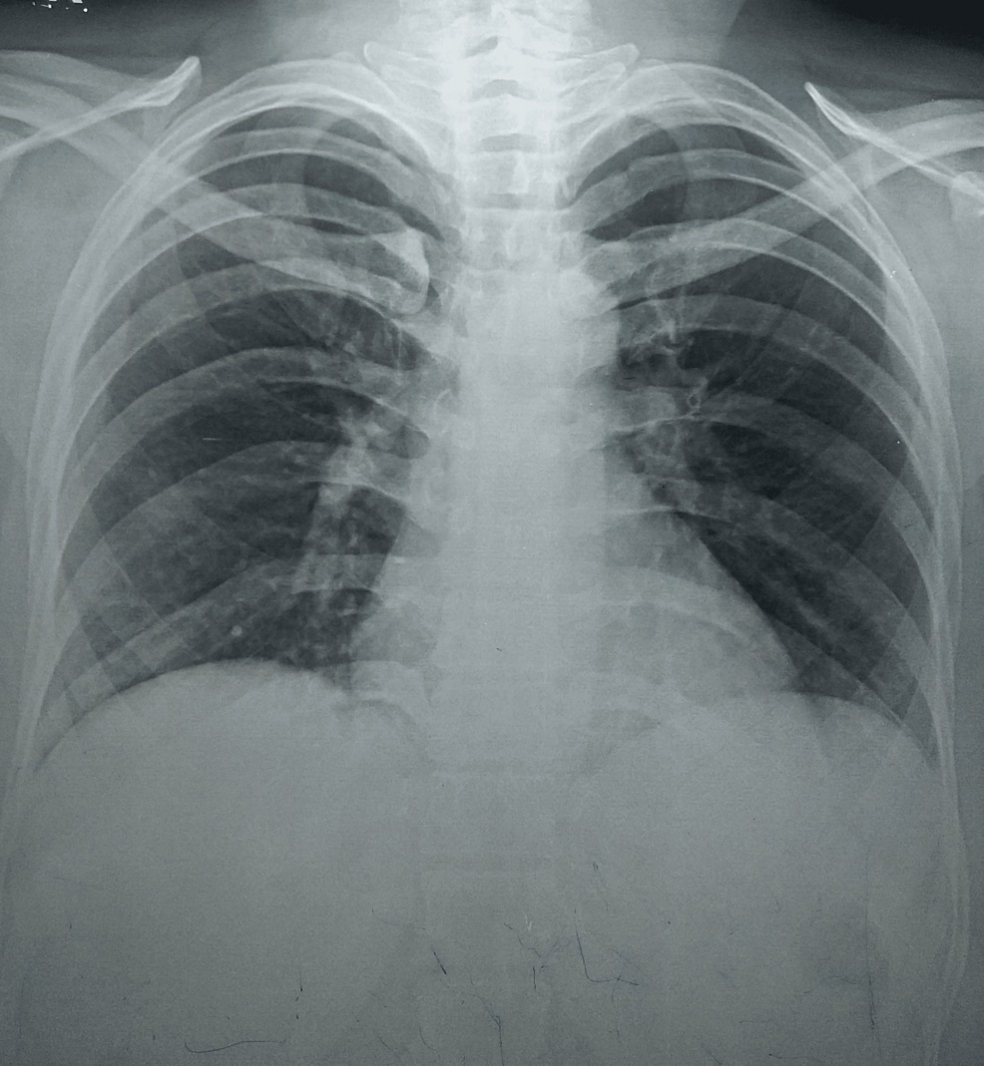
A normal chest radiograph posteroanterior (PA) view

**Figure 2:**
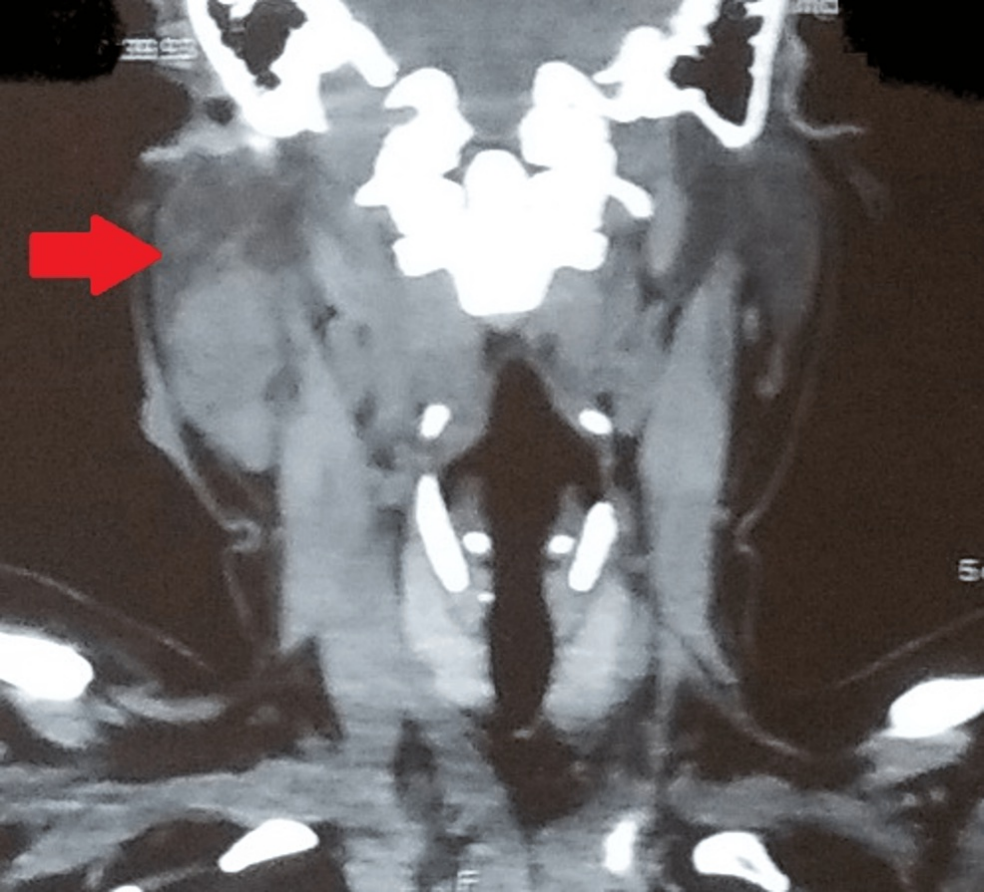
A multi-slice computed tomography (CT) of the neck (sagittal view) A multi-slice CT of the neck showing a bulky right parotid in its caudal aspect with a soft tissue density lesion within the parotid parenchyma closely abutting the sternocleidomastoid muscle

**Figure 3:**
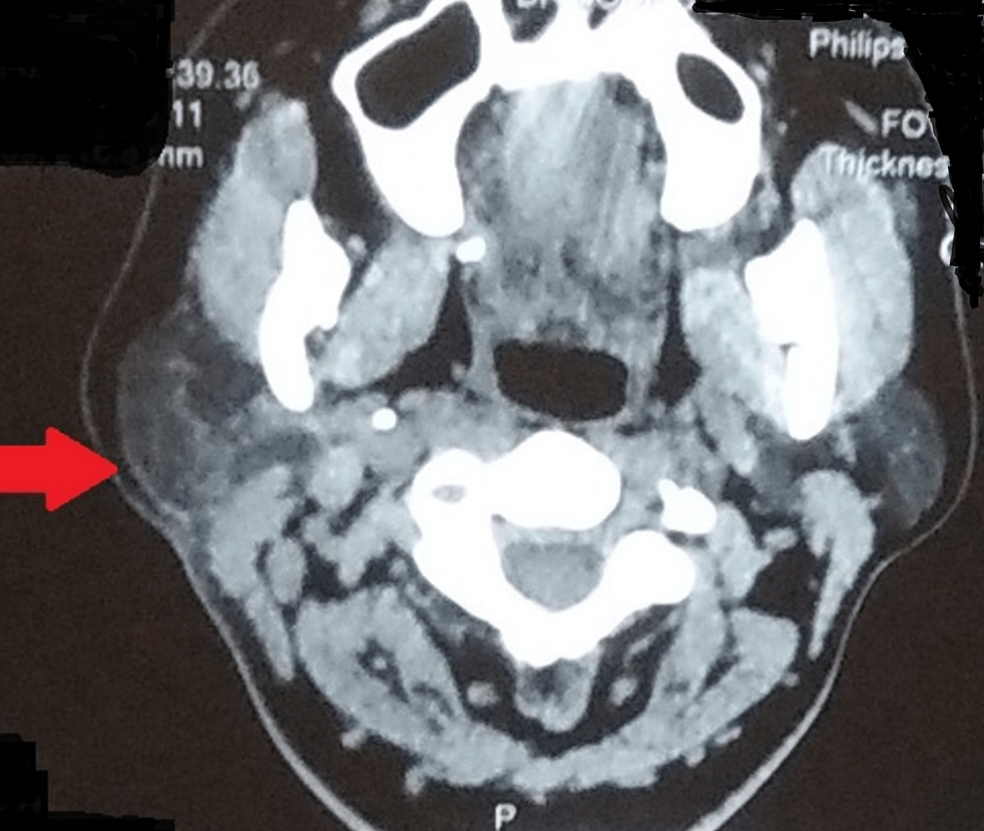
A multi-slice computed tomography (CT) of the neck (axial view) A multi-slice CT of the neck showing a small collection/abscess adjacent to the soft tissue density reaching up to the surface of the skin with a bulge

A detailed hematological workup was normal and the chest radiograph posteroanterior (PA) view was unremarkable (Figure 1). Induced sputum microscopy for acid-fast bacilli (AFB) and CBNAAT on induced sputum were negative. FNAC of the swelling was done and one more sample was sent for CBNAAT. Cytopathology was suggestive of scattered lymphocytic cells, mixed with nuclear debris and some lipid vacuoles in a necrotic background with no evidence of malignant and giant cells. Ziehl-Neelsen (ZN) staining revealed the presence of AFB. Additionally, the sample sent for the CBNAAT revealed the presence of *Mycobacterium tuberculosis* (low level) with resistance to rifampicin. Per the national guidelines, a repeat sample was obtained and a repeat CBNAAT was done with one sample sent for culture and drug susceptibility testing (DST). The results of repeat CBNAAT were the same as earlier and the culture grew *Mycobacterium tuberculosis* with no additional resistance. Ultrasonography of the right parotid was suggestive of a well-defined hypoechoic lesion with clear margins. A multi-slice CT neck was suggestive of a bulky right parotid in its caudal aspect with a soft tissue density lesion within the parotid parenchyma measuring 26 mm x 20 mm closely abutting the sternocleidomastoid muscle (Figure 2). A small collection/abscess 20 mm x 16 mm was seen adjacent to the soft tissue density reaching up to the surface of the skin with a bulge in the same (Figure 3). An enlarged adjacent right level-IB lymph node measuring 17 mm x 17 mm was also noted.

Based on the results of the investigations, a final diagnosis of primary extrapulmonary rifampicin mono-resistant tuberculosis of the parotid gland was made and per the national guidelines, a pretreatment evaluation (PTE) was done to start the second-line antitubercular drugs. Her complete blood count with platelet count, liver function tests, serum creatinine, blood urea, electrocardiogram, audiogram, thyroid profile, and fasting blood sugar levels were within normal limits. Her urine routine and microscopy were unremarkable and her urine for the pregnancy test was negative. She was HIV non-reactive and her mental health evaluation was not suggestive of any abnormal findings. A surgical intervention from a thoracic surgeon was not required as the chest radiograph was normal.

As the PTE was within normal limits, the patient was started on a WHO-recommended regimen after a discussion at the nodal drug-resistant tuberculosis center with isoniazid, pyrazinamide, ethambutol, high-dose moxifloxacin, clofazimine, ethionamide, pyridoxine, and kanamycin (Table 1).

**Table 1:** World Health Organization-recommended regimen after a discussion at the nodal drug-resistant tuberculosis center NA- Not applicable

Drug	Dose (Initial Phase) 4 months	Dose (Continuation Phase) 5 months
Isoniazid	900 mg (per oral)	NA
Pyrazinamide	2000 mg (per oral)	2000 mg (per oral)
Ethambutol	1600 mg (per oral)	1600 mg (per oral)
Moxifloxacin (High dose)	800 mg (per oral)	800 mg (per oral)
Clofazimine	200 mg (per oral)	200 mg (per oral)
Ethionamide	1000 mg (per oral)	NA
Kanamycin	1000 mg (intramuscular)	NA
Pyridoxine	100 mg (per oral)	100 mg (per oral)

Post the treatment initiation, there was significant relief to the patient with a reduction in the size of right parotid swelling and she continued to complete her full treatment of nine months with complete resolution. The whole treatment was not associated with any significant adverse drug reaction. However, a repeat CT head and neck was not done due to the non-availability of free spots and the patient’s unwillingness due to financial issues.

## Discussion

Extrapulmonary tuberculosis accounts for nearly 20% of the total tuberculosis cases [4]. This disease could manifest at different extrapulmonary sites with the most common being tuberculosis of the lymph nodes [5]. Lymph nodes of the cervical region and those around the salivary glands are commonly involved [5]. Tuberculosis of salivary glands is very rare and that of the parotid glands is the rarest of rare clinical presentations [6]. Further, rifampicin mono-resistance has never been reported in parotid glands.

The diagnosis of parotid tuberculosis is a challenging task due to the similarities in presentation with neoplasms [7]. Birkent et al. in 2008 reported that it requires a very high index of suspicion, especially in the absence of any symptoms of tuberculosis or any history of contact [4]. The rarity of this clinical entity could result in missed diagnosis and superfluous parotidectomies [6]. 

It is evident that there is a paucity of literature related to drug-resistant tuberculosis of the parotid gland and no case of primary rifampicin mono-resistance is penned down. Only one case of isoniazid-resistant tuberculosis has been reported in the literature of a 38-year-old female from the Dominican Republic who moved to the US [8]. The present case shares similarities with the case of Lee et al. in the absence of any clinical features suggestive of tuberculosis, no personal or family history of tuberculosis, no pulmonary involvement, and diagnosis based on FNAC [8]. However, the present case differs from the case of Lee et al. in the early use of techniques like the CBNAAT which resulted in timely diagnosis, the type of resistance which was rifampicin mono-resistance, and medical management instead of surgical excision/parotidectomy [8]. Also, in the case of Lee et al., the findings on the CT and FNAC were contrasted with the cytology suggestive of Warthin’s tumor but in the present case, no such ambiguity was present [8].

It is noteworthy that the patient belonged to a high-burden country and the diagnosis of primary rifampicin mono-resistance was made due to the availability of the trained staff which resulted in early CBNAAT leading to diagnosis. It also helped in avoiding unnecessary surgery which can cause facial weakness, permanent facial nerve injury, Frey syndrome, first-bite syndrome, and skin hypo-anesthesia [9,10].

The strength of this case includes a detailed history, an early CBNAAT, and efforts to rule out the differentials thereby avoiding an unnecessary parotidectomy. The results of both FNAC and CBNAAT were available simultaneously which helped in preventing the delay in treatment. The only limitation of this case is the unavailability of a repeat CT head and neck at the treatment completion and the data is from a single case; therefore, it is essential to have large-scale studies aimed at such drug resistance in isolated cases. This case would help modify the existing guidelines or form new guidelines for the management of such rare clinical presentations. It is also recommended that a very high index of suspicion is required to diagnose such cases for timely initiation of management.

## Conclusions

A case of primary rifampicin mono-resistance in the parotid gland is presented here. The early diagnosis and prompt management with a WHO-recommended regimen helped our patient recover in nine months. The case emphasizes the need for a very high degree of suspicion especially in high-burden countries to diagnose such cases. Parotid swellings masquerade as malignancies that are often misdiagnosed and result in unnecessary surgeries which has a negative impact on patients. The use of CBNAAT with FNAC, in this case, was really helpful, as the paucibacillary cases or cases where samples are difficult to obtain require both investigations to be done simultaneously; this will prevent the needle pricking multiple times and also reduce the chances of unintentional facial nerve injury. It is also evident from this case that rare presentations are common and to establish the diagnosis, there should be a detailed history and clinical examination backed by an extensive diagnostic workup. Furthermore, it is imperative to have a repeat CT head and neck in all such cases and the same should be included in the guidelines of national tuberculosis elimination programs. 

## References

[1] Yadav S (2022). Grade III severe QT prolongation in an Indian male on all-oral longer regimen for multidrug-resistant pulmonary tuberculosis: world’s first case. Cureus.

[2] (2022). World Health Organization: global tuberculosis report 2022. https://www.who.int/teams/global-tuberculosis-programme/tb-reports/global-tuberculosis-report-2022..

[3] Salaam-Dreyer Z, Streicher EM, Sirgel FA (2021). Rifampicin-monoresistant tuberculosis is not the same as multidrug-resistant tuberculosis: a descriptive study from Khayelitsha, South Africa. Antimicrob Agents Chemother.

[4] Birkent H, Karahatay S, Akcam T, Durmaz A, Ongoru O (2008). Primary parotid tuberculosis mimicking parotid neoplasm: a case report. J Med Case Rep.

[5] Hamdan AL, Hadi U, Shabb N (2002). Tuberculous parotitis: a forgotten entity. Otolaryngol Head Neck Surg.

[6] Singh D, Mishra S (2021). A rare case of parotid gland tuberculosis. Case Rep Pediatr.

[7] Holmes S, Gleeson MJ, Cawson RA (2000). Mycobacterial disease of the parotid gland. Oral Surg Oral Med Oral Pathol Oral Radiol Endod.

[8] Lee S, Chirch LM, Sosa L, Wu Q, Falcone TE (2021). Isoniazid-resistant tuberculosis of the parotid gland masquerading as a benign neoplasm. Ear Nose Throat J.

[9] Kligerman MP, Jin M, Ayoub N, Megwalu UC (2020). Comparison of parotidectomy with observation for treatment of pleomorphic adenoma in adults. JAMA Otolaryngol Head Neck Surg.

[10] Fiacchini G, Cerchiai N, Tricò D, Sellari-Franceschini S, Casani AP, Dallan I, Seccia V (2018). Frey Syndrome, first bite syndrome, great auricular nerve morbidity, and quality of life following parotidectomy. Eur Arch Otorhinolaryngol.

